# Management of Hepatitis A-induced Macrophage Activation Syndrome With Anakinra in Systemic Juvenile Idiopathic Arthritis: A Case Report

**DOI:** 10.7759/cureus.21430

**Published:** 2022-01-19

**Authors:** Deena N AlNouwaiser, Sajdi S AlMutairi, Abdullah S AlRowailey

**Affiliations:** 1 Department of Pediatrics, King Saud University Medical City, Riyadh, SAU

**Keywords:** rheumatic disease, pediatrics, systemic juvenile idiopathic arthritis, corticosteroids, anakinra, hepatitis a, macrophage activation syndrome

## Abstract

Macrophage activation syndrome (MAS) is a severe and life-threatening complication of rheumatic diseases in childhood. It is most associated with systemic juvenile idiopathic arthritis (sJIA). We present the case of a nine-year-old boy diagnosed with sJIA for six years who developed MAS triggered by hepatitis A. He was managed with anakinra and corticosteroids. Some of the clinical features of MAS occur late in the disease course, so clinicians should keep a high index of suspicion to initiate treatment early. This case highlights that anakinra and corticosteroid use in treating MAS is effective and has a good safety profile for pediatric patients.

## Introduction

Macrophage activation syndrome (MAS) is a life-threatening complication of rheumatic diseases in children. MAS identifies hemophagocytic syndrome in children with systemic juvenile idiopathic arthritis (sJIA) and other chronic rheumatic diseases [1]. MAS may develop spontaneously, as a complication of active disease, or can be induced by an infection, changes in drug therapy, or a toxic effect of medication [2]. Most cases (58% to 88.9%) have possible triggering factors prior to the onset of MAS [1]. We present the case of a nine-year-old boy with sJIA who developed MAS triggered by a hepatitis-A infection.

## Case presentation

A nine-year-old Yemeni boy with sJIA presented to our emergency department (ED) with a four-week history of jaundice, a two-week history of abdominal pain and gradual distension, and a three-month history of intermittent fever. His initial diagnosis of sJIA was made in our hospital six years prior to his current presentation based on a high-grade, six-week intermittent fever, a rash that coincided with the fever spikes, and myalgia. His care team prescribed anakinra, an interleukin-1 antagonist, to manage his sJIA. He has no family history of hemophagocytic lymphohistiocytosis (HLH), and he had not developed any severe flares requiring hospitalization since his diagnosis. However, his symptoms returned on all attempts to wean his anakinra regimen. Prior to his current admission, he was evaluated in the rheumatology clinic five months ago and was maintained on anakinra (100 mg subcutaneous injection) every other day.

Over the preceding three months, his mother recognized a suboptimal response to anakinra when they were visiting Yemen-he developed fever with rash and myalgia every one to two days. In the previous month, he developed gastroenteritis, for which he sought medical advice in Yemen and was diagnosed with hepatitis A and received supportive treatment. Several days later, his family noticed his jaundice and mild irritability. He also had tea-colored urine, decreased appetite, and subjective weight loss. He had multiple episodes of epistaxis and vomiting (no blood); however, there was no history of melena or hematemesis. 

As his symptoms worsened, his parents sought medical advice from multiple hospitals on their return to Riyadh. His workup revealed elevated liver enzymes and direct hyperbilirubinemia, and he was positive for hepatitis A. The physician in his last visit advised his parents to stop anakinra and visit a tertiary hospital when he presented to our ED. 

He was conscious and oriented on presentation to the ED, but he looked sick, deeply jaundiced, and in pain. We noted his tense, distended abdomen, tender hepatomegaly, and a palpable spleen 2 cm below the costal margin on physical examination. He had a Glasgow-coma scale (GCS) of 15/15, and his pupils were equally reactive with normal muscle tone, power, and reflexes. He showed no signs of encephalopathy. The findings from his cardiovascular, respiratory, and musculoskeletal examinations were unremarkable. He had no skin rash or lymphadenopathy. His presenting vital signs are listed in Table 1. His initial laboratory evaluations showed white blood cell (WBC), hemoglobin (Hb), and platelet counts within reference ranges. His coagulation profile was abnormal, and he had elevated liver enzymes, direct hyperbilirubinemia, and hypoalbuminemia. His immunoglobulin (Ig) M screening was positive for hepatitis A, and Epstein-Barr virus (EBV) IgG was positive. Screens for hepatitis B, hepatitis C, human immunodeficiency virus, and cytomegalovirus were negative (Table 2).

**Table 1:** Vital signs during emergency department evaluation

Assessment	Value
Temperature	36.4°C
Heart rate	120 beats/minute
Respiratory rate	20 breaths/minute
Blood pressure	100/57 mmHg
O_2_ saturation	99% on room air

**Table 2:** Laboratory evaluation results in the emergency department and day 0, day 1, and day 9 of admission Abbreviations: ALT, alanine transaminase; APTT, activated partial thromboplastin time; AST, aspartate transaminase; CMV, cytomegalovirus; CRP, C-reaction protein; EBNA, Epstein-Barr Virus Nuclear Antigen; EBV, Epstein-Barr Virus; ED, emergency department; ESR, erythrocyte sedimentation rate; HDL, high-density lipoprotein; IgG, immunoglobulin G; IgM, immunoglobulin M; INR, international normalized ratio; LDL, low-density lipoprotein; NA, not applicable; ND, not done; PT, prothrombin time; VCA, viral capsid antigen. *Ferritin values mentioned above were obtained within one to two days from the listed days. ** The values mentioned above were obtained one day after the listed day.

Analyte	ED	Day 0	Day 1	Day 9
White blood cells (x10^9^/L)	11.000	3.500	2.300	9.300
Red blood cells (x10^12^/L)	3.8	2.8	2.8	3.5
Platelets (x10^9^/L)	199	149	155	103
Hemoglobin (g/L)	10.3	7.5	7.5	10.4
ESR (mm/h)	ND	7	ND	ND
PT (s)	>90	27.2	>90	26.70
INR	>7	2.15	>7	1.99
APTT (s)	105.8	62.3	91.40	42.70
Fibrinogen (g/L)	ND	ND	0.40	ND
ALT (µkat/L)	984	>1,000	811	269
AST (µkat/L)	ND	>1,000	608	103
Albumin (g/L)	14.7	22.01	26.01	24
Direct bilirubin (µmol/L)	238.88	176.98	152.32	191
Indirect bilirubin (µmol/L)	67	56	70	91
Total bilirubin (µmol/L)	305.79	233.22	223.63	282
Glucose (mmol/L)	5	11.02	11.71	8.29
Potassium (mmol/L)	3.1	2.6	2.7	5
Sodium (mmol/L)	135	139	140	136
Ferritin (µg/L) *	>16,500	ND	1,413	335
CRP (mg/L) **	ND	5.770	2.360**	ND
Procalcitonin (µg/L)**	ND	ND	0.43**	ND
HDL (mmol/L)	ND	2.10	ND	ND
LDL (mmol/L)	ND	1.34	ND	ND
Triglycerides (mmol/L)	ND	0.91	ND	ND
Blood cultures	3 Negatives	Negative	N/A	N/A
Urine culture	Negative	Negative	N/A	N/A
Hepatitis A IgM	Positive	N/A	N/A	N/A
EBV IgG VCA	Positive	N/A	N/A	N/A
EBV IgG EBNA	Positive	N/A	N/A	N/A
EBV IgM VCA	Negative	N/A	N/A	N/A
CMV IgM	Negative	N/A	N/A	N/A

He was admitted to the pediatric intensive care unit (PICU) as a case of acute liver failure. Upon admission to the PICU, the patient received 1 mg/kg of albumin, vitamin K, one dose of factor VII prior to femoral line insertion, norepinephrine 0.03 µg, and one dose of methylprednisolone 1 mg/kg. Later that day, he became irritable, drowsy, difficult to arouse, tachypneic, and hypotensive. His second laboratory evaluation revealed a significant drop in WBC, Hb, and platelet counts and worsening liver enzymes and liver function tests (Table 2). According to his laboratory results and clinical presentations, he received a diagnosis of MAS triggered by hepatitis A infection. We started treatment immediately with one dose of intravenous immunoglobulin (IVIG) of 2 mg/kg along with intermittent IV methylprednisolone 30 mg/kg/day for three days and vitamin K. Anakinra continued to be withheld. We also administered a dose of epinephrine 0.03 µg/kg because his blood pressure was outside of the reference range.

On the second day of admission, his consciousness level decreased. He was difficult to arouse and irritable when woken up. He was sleeping and unresponsive to painful stimuli and had mild lower limb rigidity when examined. His GCS was 7/15. He developed bilateral symmetrical hyperreflexia in both upper and lower limbs and clonus. A new set of laboratory values were obtained (Table 2). EEG and CT findings were unremarkable. We resumed his IV anakinra 100 mg every six hours and extended his intermittent IV methylprednisolone to five days. He continued 2 mg/kg IV methylprednisolone divided into two doses daily and vitamin K. The patient did not significantly improve clinically until the ninth day of treatment when he recovered completely. He was alert and oriented, and he was able to walk around and feed orally. He was shifted to the general ward, maintained his stability, and then discharged in good condition. At his one-month follow-up evaluation, his condition was stable.

## Discussion

MAS is a severe life-threatening complication of rheumatic diseases in childhood and a secondary form of HLH [3,4]. MAS is characterized by the uncontrolled activation and proliferation of T lymphocytes and macrophages, resulting in the overproduction of inflammatory cytokines [4,5]. Clinically, MAS is characterized by unremitting fever, pancytopenia, hepatosplenomegaly, elevated liver enzymes, neurologic symptoms, coagulation abnormalities, and hyperferritinemia [4-6]. Due to the clinical heterogenicity of the condition, the Histiocyte Society suggests preliminary criteria for the diagnosis of secondary HLH, including clinical, laboratory, and sometimes histopathological findings [4,7]. Since our patient had classic clinical and laboratory findings, we did not conduct a bone marrow biopsy due to coagulopathy and clear evidence of HLH.

In low-income countries, hepatitis A is still a highly prevalent disease [4]. Although acute infections with hepatitis A virus in children are self-limited, 0.1% of patients progress to fulminant hepatic failure that could result in death, liver transplantation, or more rarely, spontaneous recovery as in our patient [4]. 

Infections caused by the herpes group of viruses, particularly EBV, are well-known triggers for MAS in sJIA patients [6]. However, there are only six reported hepatitis A virus-induced MAS cases in children [4-6]. Four of the cases were previously healthy, while only two were previously known cases of sJIA [4-6]. This case represents the first reported case of MAS induced by hepatitis-A in a patient with sJIA in Saudi Arabia.

Although the mechanism of MAS is still poorly understood, cytokine over-production plays a significant role [4]. Since there are no standardized treatment protocols for MAS, most cases in the literature, including the previously mentioned cases, were treated according to the HLH-2004 treatment protocol with cyclosporine A, dexamethasone, and etoposide [7,8]. Due to the potential cytotoxic effect of cyclosporine A, other treatment protocols with better safety profiles have been suggested [8]. Anticytokine therapies such as etanercept and anakinra have been recently used to treat MAS, and they have been effective in inducing and maintaining remission with fewer adverse effects [8]. Moreover, there are several reported cases that were refractory to the use of cyclosporine A and glucocorticoids but were managed successfully with Anakinra [9]. We managed our patient with one dose of 2-mg/kg IVIG and intermittent IV methylprednisolone 30 mg/kg/day for five days. We also administered 100 mg IV anakinra every six hours. On the ninth day of treatment, the patient completely clinically recovered regarding orientation, feeding, and ability to move. The patient did not have any treatment-related adverse effects.

## Conclusions

We discussed the case of a nine-year-old boy with sJIA who developed hepatitis A-induced MAS. Following treatment with IVIG, corticosteroids, and anakinra, the patient had complete resolution of his clinical symptoms and laboratory abnormalities. This case highlights that anakinra and corticosteroid use in treating MAS is effective with a good safety profile for pediatric patients.
